# Multiloculated thoracoabdominal tuberculosis: A radiological presentation of disseminated tuberculosis

**DOI:** 10.1016/j.radcr.2024.09.041

**Published:** 2024-09-24

**Authors:** Muhammad Bilal Ibrahim, Reyan Hussain Shaikh, Arshia Jahangir, Ali Husnain Khan, Hiba Noor

**Affiliations:** a Medical Graduate, Aga Khan University Hospital, Karachi 74800, Pakistan; b Medical College, Aga Khan University, Karachi 74800, Pakistan; c Medical College, Sahara Medical College, Narowal, Pakistan; d Medical College, Fatima Jinnah Medical University, Lahore

**Keywords:** Hepatic Tuberculosis, Disseminated Tuberculosis, Mycobacterium tuberculosis, Subligamentous spread, Contiguous spread, Hepatic abscess

## Abstract

Tuberculosis is more frequently found among high-risk populations in the United States. It has a challenging diagnosis since it can present with diverse organ involvement that may delay the diagnosis. This is especially true regarding hepatic tuberculosis, with prevalence varying in each study but highly suggestive of underdiagnosis. An 18-year-old male with high-risk exposure to multidrug-resistant tuberculosis presented with fever, night sweats, weight loss, and cough. Imaging revealed a right lung cavitary mass with bilateral pulmonary nodules, right pleural nodular thickening traversing diaphragm extending to the liver with subcapsular hepatic lobulated hypodensities. MRI showed spinal involvement consistent with Pott's disease. It is important to consider hepatic tuberculosis in differential diagnoses for a hepatic lesion, allowing early detection and treatment to optimize patient outcomes.

## Introduction

Tuberculosis (TB) has been recognized as the second most fatal infectious disease after Coronavirus disease (COVID-19) by the World Health Organization (WHO), contributing to a total of 1.3 million deaths worldwide in 2022 [1]. Despite its higher prevalence in developing countries, the increase in emigration, and accelerated deterioration in immunity with Human Immunodeficiency Virus (HIV) co-infection [2], TB poses significant health challenges in developed countries such as the United States (US) [3].

TB primarily presents as a pulmonary disease, however about 20-25% of the cases have extrapulmonary involvement [4]. In particular, hepatic involvement is a rare occurrence (1%) [5]. Hepatic tuberculosis (HTB) is a diagnostic challenge; therefore, it often remains undetected and is usually diagnosed on autopsy or surgery [6]. This is mainly because the clinical features associated with HTB are nonspecific and imaging usually hints towards hepatic neoplasms [6]. The inconclusive results from the imaging modalities [7], along with nonspecific symptoms like fever, weight loss, and ascites lead to potential misdiagnosis and further complications [5,8,9].

## Objective

Given the complexity associated with the timely diagnosis of HTB, we present a case report of an 18-year-old male who presented with cough, weight loss, and night sweats in a developed country that is characterized by low TB incidence rates. After extensive imaging and diagnostic tests, a rare radiological presentation of disseminated TB led to the identification of HTB as the underlying condition. This case is unique given the scarcity of literature on TB resulting in multiloculated contiguous spread from lungs, in contrast to the more commonly reported single hepatic abscess.

## Case report

An 18-year-old male of native Spanish origin, presented to the emergency department with 2 months long history of sore throat, night sweats, dry cough, loss of appetite, undocumented fever spikes associated with chills, and undocumented weight loss.

His medical history revealed that he had recently been exposed to TB through his roommate who had tested positive for Isoniazid resistant TB some time ago and was receiving anti-tuberculosis therapy (ATT). He also mentioned that there were multiple people living in the same dormitory. There was no recent history of incarceration or travel to endemic countries. Further details on the patient's social history were not obtained as the interpreter faced difficulties with the patient's Guatemalan dialect Kekchi. Other symptoms reported by the patient during the history included back pain near the scapular region. The patient had a positive addiction history, with 1-2 cigarettes every day for the past 2 weeks and 9 drinks a day. However, the dialect served as a major hurdle in obtaining a more thorough addiction history.

Upon physical examination, he appeared distressed and lean, but no scleral icterus was observed. The auscultation of the chest revealed decreased breath sounds bilaterally at the lung bases, with inspiratory wheeze in the right upper zone. The spine was tender on palpation, while no organomegaly was found on abdominal examination. The rest of the physical examination was unremarkable.

**Fig. 1:**
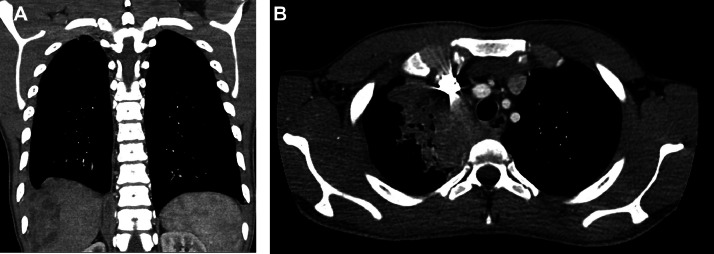
Chest CT scan, in coronal (A) and sagittal (B) sections. Imaging revealing large right upper lung cavitary mass with bilateral pulmonary nodules and masses. Nodularity of the right pleura is also seen, representing possible metastatic disease or complex pleural collection in the setting of TB. Adjacent subcapsular lobulated lesions were noted in the right hepatic lobe.

Chest X-ray revealed right upper lung consolidative airspace opacity, and hazy airspace opacity in the right inferior lung. Small right pleural effusion and adjacent subcapsular lobulated lesions were also noted in the right hepatic lobe, concerning TB. Computed Tomography (CT) scan of the chest revealed a large right upper lung cavitary mass and bilateral pulmonary nodules and masses, along with nodularity of the right pleura indicating malignancy or TB (Fig. 1).

Based on the symptoms, exposure history, and imaging findings, a case of pulmonary TB was suspected. Consequently, sputum acid-fast bacteria (AFB) stains and *Mycobacterium tuberculosis* polymerase chain reaction (PCR) tests were performed. The patient was given precautions regarding the airborne spread of the disease and asked to settle in quarantine. Moreover, appropriate medications were started, which included 500 mg Levofloxacin (Levofloxacin, Major Pharmaceuticals, US), 1200 mg Ethambutol (Ethambutol Hydrochloride, American Health Packaging, US), 1500 mg Pyrazinamide (Pyrazinamide Tablets, Novitium, US), 600 mg Rifampin (Rifampin Capsules, American Health Packaging, US), and 50 mg oral vitamin B6 (Vitamin B-6 (from Pyridoxine Hydrochloride), GenDose Pharmaceuticals, US) daily.

Results from the patient's laboratory work-up are summarized in Table 1.

**Table 1:** Laboratory results.

Laboratory tests	Units	Result	Reference range
Alkaline phosphatase (ALP)	U/L	157	20-120
Gamma-glutamyl transferase (GGT)	U/L	257	3-60
Aspartate transaminase (AST)	U/L	47	0-40
Alanine transaminase (ALT)	U/L	43	5-35
Lactate Dehydrogenase (LDH)	U/L	246	85-210
Albumin	g/L	38	38-52
Hemoglobin (HGB)	mmol/L	7.4	8.0-10.4
Platelet count (PLT)	x 10^9/L	630	161-369

Laboratory work-up revealed deranged liver function tests (LFTs), consistent with the cholestatic pattern.

The patient tested negative for HIV and COVID-19. These liver enzymes and imaging raised suspicion of possible hepatic involvement of TB, however, to rule out malignancy a CT scan was performed. The presence of spinal tenderness on physical examination raised suspicion of Pott's disease, hence Magnetic Resonance Imaging (MRI) was also ordered.

**Fig. 2:**
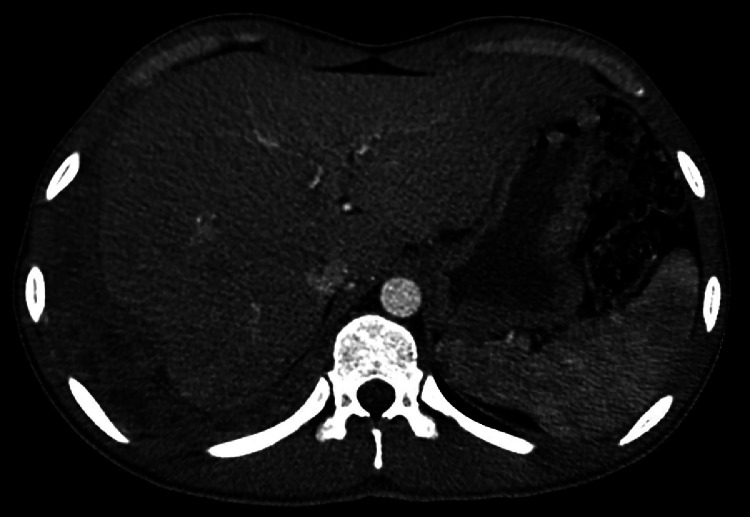
CT scan of the abdomen. Imaging revealing right pleural thickening with loculated low-attenuation areas and rim enhancement, extending to the diaphragm and the liver capsule, suggestive of multiloculated abscess/empyema from tuberculosis.

**Fig. 3:**
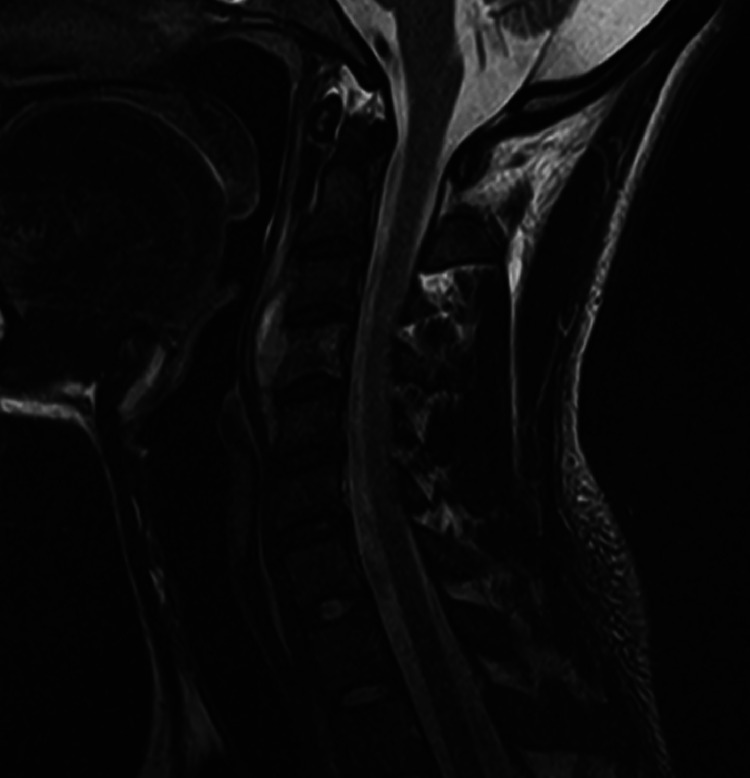
MRI of the cervical spine. Enhancing soft tissue noted epicentered along the anterior margin of C4 vertebral body with loss of anterior cortex and mild marrow enhancement, extending both superiorly and inferiorly along the C3 and C5 vertebra. Findings suggest subligamentous spread of TB, characterised by relative sparing of the disc spaces.

Sputum PCR and AFB smear were positive for *M. tuberculosis*, hence confirming the diagnosis of pulmonary TB. The liver CT returned with abnormal findings, with heterogeneously attenuated lobulated right pleural density that extended inferior to the diaphragm and abutted the right liver capsule. Multiloculated hepatic abscess was also noticed (Fig. 2). This was related to TB, and malignancy was fairly ruled out. There was no evidence of hepatic dysfunction or cirrhosis. The spinal MRI revealed enhancing soft tissue epicentered along the anterior margin of the C4 vertebral body with loss of anterior cortex and mild marrow enhancement. It extended both superiorly and inferiorly along the C3 and C5 vertebrae (Fig. 3). These imaging characteristics were compatible with the subligamentous spread of TB infection. There was relative sparing of disc spaces—a characteristic of TB. Multifocal necrotic lesions were noted along the right paraspinal soft tissues, with no extension of disease into the spinal canal.

The patient's extrapulmonary TB was managed similarly to the pulmonary TB, with continuation of the ATT for 9 months. The first 10 doses of ATT were administered to the patient in the hospital, after which an improvement in LFTs was observed, as shown in Table 2. Furthermore, Interventional Radiology (IR)-guided perihepatic/subpleural abscess drainage was performed.

**Table 2:** LFTs after treatment initiation.

Laboratory tests	Units	Result	Reference range
Alkaline phosphatase (ALP)	U/L	107	20-120
Gamma-glutamyl transferase (GGT)	U/L	188	3-60
Aspartate transaminase (AST)	U/L	31	0-40
Alanine transaminase (ALT)	U/L	48	5-35
Lactate Dehydrogenase (LDH)	U/L	102	85-210

Improvement in LFTs was observed after first 10 doses as the patient started ATT.

The patient was then discharged for outpatient follow-up. In a follow-up visit 2 months later, the patient expressed improvement in symptoms, including resolution of cough, improved appetite, less fatigue, and no night sweats or fever. However there was no significant weight gain yet. Unfortunately, as the patient was an undocumented immigrant, he relocated to a different state, resulting in a loss of follow-up at our institution. Nonetheless, subsequent reports from the tuberculosis surveillance team indicated that the patient had clinically improved, secured employment in a different state, and successfully completed the prescribed treatment.

## Discussion

The incidence of TB, and hence extrapulmonary TB has increased in recent years, owing to the increase in HIV/Acquired Immune Deficiency Syndrome (AIDS) cases, multidrug resistance, and intravenous (IV) drug abuse [10]. Moreover, over the past many years, the US has received large migration cohorts, from Europe until the 1970s, and now majorly from Latin America and Asia. The non-US born individuals have a high burden of latent tuberculous infection (LTBI) that has the potential for late reactivation many years after the initial infection [11].

The most common site of *M. tuberculosis* spread is to the abdominal organs, accounting for 5% of all TB cases worldwide [[12], [13], [14]]. Particularly, a rare manifestation is liver involvement, which accounts for only 1% of the total active TB cases [5]. On the other hand, HTB occurs in 50%-80% of patients who die from pulmonary TB [15].

The rich blood supply of the liver facilitates the formation of granulomas, especially near the portal tract with little to no hampering of the hepatic function, therefore these patients clinically present as asymptomatic or with minimal symptoms [16]. However, the rare nature of such cases is primarily due to the low oxygen tension in liver, which is not ideal for the growth of *Mycobacterium* due to its obligate aerobic nature [17].

HTB has been classified in the literature on various bases, but here we focus primarily on the involvement of liver in TB in 3 forms—miliary TB, granulomatous hepatitis TB, and localized hepatobiliary TB [18]. The miliary TB refers to the systemic spread of bacteria and seeding into different organs, and this form accounts for about 79% of the total reported HTB cases [5]. On the other hand, the local HTB cases account for 21% of the reported cases, with the hepatobiliary occurrence being rare [5]. In our case, the patient belonged to the first category, that is, the miliary form of hepatic TB.

The tubercles formed in the miliary and local HTB are also distinct, with the miliary form primarily having tubercles less than 2mm in diameter along with diffuse seeding of the liver [19]. In contrast, the local HTB presents tubercles greater than 2mm in diameter situated near the portal tracts [19]. In addition to this, the arrival of the bacteria to the liver in the miliary form is via the hepatic artery and in case of local HTB it is via the portal vein [19].

HTB presents clinically with nonspecific symptoms, making its diagnosis difficult [7]. In most studies, the symptoms include fever, malaise, abdominal pain, weight loss, and respiratory symptoms [5,8,9]. Upon physical examination, the most consistent sign is hepatomegaly, which is present in 96% of the patients [17]. Splenomegaly, ascites, and jaundice are also observed in some patients [5,20]. In our case, none of these symptoms were observed. Patients with miliary HTB, as in our case, usually have respiratory symptoms like cough with or without sputum, whereas the other 2 forms commonly present with abdominal pain and may have jaundice [8,19,21].

Laboratory findings usually show elevated liver enzymes, particularly ALP, GGT, and transaminases [22]. In this patient, these lab parameters were raised. If pulmonary TB is suspected, as in our case, the most common tests include sputum AFB smear microscopy and culture, along with PCR testing.

Different imaging modalities reveal different findings. Despite the lack of diagnostic specificity, plain X-ray and ultrasound are still used as first-line investigations [23]. A chest X-ray may indicate pulmonary TB, which can aid in the diagnosis [24]. Ultrasound usually reveals round, hypoechoic lesions with no specificity for detecting HTB, hence is not a recommended test [25,26].

Abdominal CT is the preferred diagnostic test. In miliary TB, CT typically reveals multiple micronodules throughout the liver as opposed to local hepatic TB which reveals a large solitary or a few nodules [25,27]. In a liver tuberculoma, the caseation necrosis appears as nonenhancing at the center, as opposed to the outer granulation tissue that exhibits enhancement at the periphery [28]. In contrast to this, our case presented with TB as multiloculated hepatic abscess, via contiguous spread from the lungs, a finding that has not been seen previously.

On MRI, macronodular tubercular lesions are characterized by hypointensity on T1-weighted images [23,27]. The variability in the intensity is dependent on the stage of the disease, with a peripheral hypointense rim on T2-weighted images [23,28].

The most accurate test to be performed whenever feasible is lung biopsy, along with histopathological examination, which typically reveals caseating granulomas in the case of HTB [29]. In our setting, a biopsy was not performed as the diagnosis could be confirmed using imaging and laboratory results, without the need for an invasive procedure.

Once diagnosed, HTB is treated with ATT, usually continued for 6-12 months [30]. However, some studies recommend continuing ATT for at least 1 year [29]. It is noteworthy that many drugs are hepatotoxic and may cause further damage to the liver [29], hence close monitoring of the patients and their liver enzymes is essential throughout the course of medication.

It is also important to recognize that due to the nonspecific presentation of HTB, it is often overlooked and misdiagnosed as an abscess, cyst, or benign tumor [31]. In regions where TB is prevalent, this misdiagnosis is further augmented by the simultaneous occurrence of chronic liver disease [32]. Moreover, it is crucial to recognize both infectious and noninfectious conditions that can result in hepatic granulomas, whether caseating or noncaseating. These include leprosy, sarcoidosis, Hodgkin's disease, brucellossis, infectious mononucleosis, inflammatory bowel disease, drug-induced liver injury, and syphilis [29]. This comprehensive understanding aids in the differential diagnosis of HTB [31]. *Mycobacterium* can also make its way to other organs, including the bones, with the involvement of the spine in about half of the cases [33]. This was also seen in our case showing the extent to which the organism had spread throughout the body. The spinal involvement was suspected due to the tenderness on examination, thus stressing the importance of a thorough physical examination.

## Conclusion

This case highlighted the rare radiological presentation of disseminated TB, where HTB manifested as contiguous multiloculated hepatic abscess. It also highlights 2 important aspects: firstly, HTB must be considered in the differential diagnosis of a hepatic lesion, even in a developed country such as the US where it may usually be less common. Secondly, it serves as a reminder that TB is still a reality in the developed countries. It is crucial to consider the socioeconomic background of the patients and use diagnostic clues for a prompt and accurate diagnosis. The spread of a communicable and a potentially lethal disease like TB can be limited if the diagnosis is done timely.

## Patient consent

We obtained written, informed consent for publication of this case from the patient.

## References

[1] World Health Organization. Tuberculosis. 2023. Available from: https://www.who.int/news-room/fact-sheets/detail/tuberculosis. Accessed April 13, 2024.

[2] Bruchfeld J., Correia-Neves M., Kallenius G. (2015). Tuberculosis and HIV coinfection. Cold Spring Harbor Perspectives in Medicine.

[3] Stojkovic M, Müller J, Junghanss T, Weber TF (2018). Radiological diagnoses in the context of emigration: infectious diseases. Rofo.

[4] Ramírez-Lapausa M., Menéndez-Saldaña A., Noguerado-Asensio A. (2015). Tuberculosis extrapulmonar, una revisión. Revista espanola de sanidad penitenciaria.

[5] Hickey A.J., Gounder L., Moosa M.Y.S., Drain P.K. (2015). A systematic review of hepatic tuberculosis with considerations in human immunodeficiency virus co-infection. BMC Infectious Diseases.

[6] Oliva A., Duarte B., Jonasson O., Nadimpalli V. (1990). The nodular form of local hepatic tuberculosis: a review. Journal of Clinical Gastroenterology.

[7] Kakkar C., Polnaya A.M., Koteshwara P., Smiti S., Rajagopal K.v., Arora A. (2015). Hepatic tuberculosis: a multimodality imaging review. Insights into Imaging.

[8] Tai W.C., Kuo C.M., Lee C.H., Chuah S.K., Huang C.C., Hu T.H. (2008). Liver tuberculosis in Southern Taiwan: 15-years clinical experience. Journal of Internal Medicine of Taiwan.

[9] Wang J.Y., Hsueh P.R., Wang S.K., Jan I.S., Lee L.N., Liaw Y.S. (2007). Disseminated tuberculosis: a 10-year experience in a medical center. Medicine.

[10] Wu Z., Wang W.L., Zhu Y., Cheng J.W., Dong J., Li M.X. (2013). Diagnosis and treatment of hepatic tuberculosis: report of five cases and review of literature. International Journal of Clinical and Experimental Medicine.

[11] Menzies N.A., Hill A.N., Cohen T., Salomon J.A. (2018). The impact of migration on tuberculosis in the United States. International Journal of Tuberculosis and Lung Disease.

[12] Burrill J., Williams C.J., Bain G., Conder G., Hine A.L., Misra R.R. (2007). Tuberculosis: a radiologic review. Radiographics.

[13] Sharma S.K., Mohan A. (2004). Extrapulmonary tuberculosis. Indian Journal of Medical Research.

[14] Rathi P., Gambhire P. (2016). Abdominal tuberculosis. Journal of Association of Physicians of India.

[15] Hussain W., Mutimer D., Harrison R., Hubscher S., Neuberger J. (1995). Fulminant hepatic failure caused by tuberculosis. Gut.

[16] Ch’ng L.S., Amzar H., Ghazali K.C., Siam F. (2018). Imaging appearances of hepatic tuberculosis: experience with 12 patients. Clinical Radiology.

[17] Husain M., Khan S., Hassan M.J. (2015). Hepatic tuberculosis mimicking metastasis in a case of carcinoma sigmoid colon. Journal of Laboratory Physicians.

[18] Alvarez S.Z. (1998). Hepatobiliary tuberculosis. Journal of Gastroenterology and Hepatology (Australia).

[19] HERSCH C. (1964). Tuberculosis of the liver. A study of 200 cases. South African Medical Journal = Suid-Afrikaanse Tydskrif Vir Geneeskunde.

[20] Küçükmetin N.T., Ince Ü., Çiçek B., Akman H., Bozta̧s G., Tözün N. (2014). Isolated hepatic tuberculosis: a rare cause of hepatic mass lesions. Turkish Journal of Gastroenterology.

[21] Kok K.Y.Y., Yapp S.K.S. (1999). Isolated hepatic tuberculosis: report of five cases and review of the literature. Journal of Hepato-Biliary-Pancreatic Surgery.

[22] Das C.J., Rednam N., Vora Z., Aggarwal A., Chandrashekhara S.H., Kundra V. (2023). Abdominal visceral tuberculosis: a malignancy mimic. Abdominal radiology.

[23] Sharma R., Dey A.K., Mittal K., Udmale P., Singh U., Mitkar S. (2015). Hepatic tuberculosis mimicking biliary cystadenoma: a radiological dilemma. Case Reports in Surgery.

[24] Arora R., Sharma A., Bhowate P., Bansal V., Guleria S., Dinda A. (2008). Hepatic tuberculosis mimicking Klatskin tumor: a diagnostic dilemma. Indian Journal of Pathology and Microbiology.

[25] Levine C. (1990). Primary macronodular hepatic tuberculosis: US and CT appearances. Gastrointestinal Radiology.

[26] Cao B.S., Li X.L., Li N., Wang Z.Y. (2010). The nodular form of hepatic tuberculosis: contrast-enhanced ultrasonographic findings with pathologic correlation. Journal of Ultrasound in Medicine.

[27] Yu R.S., Zhang S.Z., Wu J.J., Li R.F. (2004). Imaging diagnosis of 12 patients with hepatic tuberculosis. World Journal of Gastroenterology.

[28] Kawamori Y., Matsui O., Kitagawa K., Kadoya M., Takashima T., Yamahana T. (1992). Macronodular tuberculoma of the liver: CT and MR findings. American Journal of Roentgenology.

[29] Parsak C.K., Hanta I., Aslan A., Alabaz O. (2008). Isolated hepatic tuberculosis presenting as cystic-like and tumour-like mass lesions. Case reports in gastroenterology.

[30] American Thoracic Society (2003). CDC, and infectious diseases society of America, “treatment of tuberculosis,”. MMWR—Recommendations and Reports.

[31] Koh Y.D.I., Leow W.-Q. (2023). A single hepatic mass with two tales: hepatic tuberculosis and hepatocellular carcinoma. Journal of Liver Cancer.

[32] Forgione A., Tovoli F., Ravaioli M., Renzulli M., Vasuri F., Piscaglia F. (2021). Contrast-enhanced ultrasound LI-RADS LR-5 in hepatic tuberculosis: case report and literature review of imaging features. Gastroenterology Insights.

[33] Kumar K. (2016). Spinal tuberculosis, natural history of disease, classifications and principles of management with historical perspective. European Journal of Orthopaedic Surgery and Traumatology.

